# Therapy-related clonal cytopenia as a precursor to therapy-related myeloid neoplasms

**DOI:** 10.1038/s41408-022-00703-8

**Published:** 2022-07-08

**Authors:** Mithun Vinod Shah, Abhishek A. Mangaonkar, Kebede H. Begna, Hassan B. Alkhateeb, Patricia Greipp, Ahmad Nanaa, Michelle A. Elliott, William J. Hogan, Mark R. Litzow, Kristen McCullough, Ayalew Tefferi, Naseema Gangat, Mrinal M. Patnaik, Aref Al-Kali, Rong He, Dong Chen

**Affiliations:** 1 Division of Hematology, Mayo Clinic, Rochester, MN USA; 2 Division of Hematopathology, Mayo Clinic, Rochester, MN USA; 3 John H. Stroger, Jr. Hospital of Cook County, Chicago, IL USA

**Keywords:** Cancer, Haematopoietic stem cells

## Abstract

Therapy-related myeloid neoplasms (t-MN) are aggressive leukemia that develops as a complication of prior exposure to DNA-damaging agents. Clonal cytopenia of undetermined significance (CCUS) is a precursor of de novo myeloid neoplasms. Characteristics of CCUS that develop following cytotoxic therapies (therapy-related clonal cytopenia, t-CC) and outcomes following t-CC have not been described. We identified 33 patients with t-CC and compared to a cohort of the WHO-defined t-MN (*n* = 309). t-CC had a distinct genetic and cytogenetic profile: pathogenic variants (PV) in *TET2* and *SRSF2* were enriched in t-CC, whereas *TP53* PV was more common in t-MN. Ten (30%) t-CC patients developed a subsequent t-MN, with a cumulative incidence of 13%, 23%, and 50% at 6 months, 1, and 5 years, respectively. At t-MN progression, 44% of evaluable patients had identifiable clonal evolution. The median survival following t-CC was significantly superior compared all t-MN phenotype including t-MDS with <5% bone marrow blasts (124.5 vs. 16.3 months, *P* < 0.001) respectively. The presence of cytogenetic abnormality and the absence of variants in *DNMT3A*, *TET2*, or *ASXL1* (*DTA*-genes) were associated with a higher likelihood of developing a subsequent t-MN and an inferior survival. We describe a putative precursor entity of t-MN with distinct features and outcomes.

## Introduction

With the widespread adaptation of sequencing techniques, precursor states of myeloid neoplasms such as clonal hematopoiesis of indeterminate potential (CHIP), idiopathic cytopenia of undetermined significance (ICUS) or clonal cytopenia of undetermined significance (CCUS) are recognized. CHIP is defined as the presence of expanded clonal blood cells carrying one or more somatic mutations, in the absence of any detectable hematological abnormalities. CCUS is characterized by unexplained cytopenia of cytopenia in the context of clonal hematopoiesis, but in the absence of known hematological malignancies. Finally, the presence of unexplained cytopenia without the evidence of clonality is defined as ICUS. While all these entities are associated with a higher risk of subsequent hematological malignancies, their malignant potential is diverse [1, 2].

Therapy-related myeloid neoplasms (t-MN) develop as a complication of prior DNA-damaging therapies including chemotherapy, radiation, stem cell transplantation (SCT), or immunosuppressive therapies (IST) for autoimmune diseases (AID). t-MN are aggressive neoplasms with overall survival of approximately 1 year from diagnosis, regardless of the therapies employed [3, 4].

In the context of DNA-damaging therapy, the impact of coexistent clonal hematopoiesis (CH) is context dependent. For example, in lymphoma and solid tumors patients, CH increases the risk of future t-MN [5, 6]. In contrast, the presence of CH did not predict a higher risk of t-MN in multiple myeloma patients undergoing autologous SCT [7]. Moreover, the therapeutic modality as well as different therapeutic classes have a distinct pattern of CH [8]. Combined how these host-related and external forces shape the clonal evolution leading to leukemic transformation is not known.

While studying t-MN patients, we encountered patients who had received DNA-damaging therapy and developed unexplained cytopenia with clonal abnormality, without morphological evidence of a myeloid neoplasm (i.e., CCUS). As a vast majority of patients in the CHIP, ICUS, or CCUS cohorts did not receive prior DNA-damaging therapies [2, 9–11], the significance of CCUS following such therapies and its outcomes is not known. We hypothesized that clonal cytopenia following DNA-damaging therapy (therapy-related clonal cytopenia or t-CC) is a distinct entity from the WHO-defined t-MN. To test this hypothesis, we analyzed clinicopathological characteristics and outcomes of t-CC as well as the risk factors for developing a subsequent t-MN.

The emergence of any cytogenetic abnormalities following DNA-damaging therapy, raises the concern for a t-MN. In a cohort of patients exposed to prior DNA-damaging therapy, 46% patients with the deletion of chromosome 7q (del 7q) progressed to t-MN. Thus, while del 7q was associated with a very high risk of progression to t-MN; it—by itself—did not define t-MN [12]. This is in contrast to the *de novo* context, wherein unexplained cytopenia in the presence of MDS-defining cytogenetic abnormality would be diagnosed as myelodysplastic syndrome, unclassifiable (MDS-U) [13, 14]. Therefore, we studied outcomes of following the emergence of MDS-defining cytogenetic abnormalities and compared with t-CC.

## Methods

### Patient cohort

Following the institutional review board approval, we conducted a retrospective review of all adult patients treated at Mayo Clinic. After obtaining informed consent, we defined t-CC using the following criteria: (i) history of exposure to DNA-damaging agents in the form of chemotherapy, radiation, autologous SCT for non-myeloid diseases, or IST; (ii) unexplained cytopenia persisting ≥4 months; (iii) evidence of clonality using cytogenetic analysis or next-generation sequencing (NGS); and (iv) no morphological evidence of a myeloid neoplasm. t-MN was defined using the WHO guideline [13]. t-CC patients that subsequently developed t-MN were included in the t-CC cohort for the purpose of this analysis. Finally, we identified patients with prior exposure DNA-damaging therapy and unexplained cytopenia that had no morphological evidence of a myeloid neoplasm but had at least one MDS-defining cytogenetic abnormality (referred to as t-MDS (cyto). According to the WHO guidelines [13], these patients were classified as t-MN and their outcome was compared to t-CC and other t-MN phenotypes.

### Clinicopathological characteristics

Demographic and clinical characteristics including age at the time of primary condition, sex, DNA-damaging therapies received, and hematological parameters were abstracted. All available bone marrow biopsies were re-reviewed by 2 hematopathologists independently (R.H. and D.C.) to exclude t-MN. Diagnostic and therapeutic decisions were made per treating physician’s discretion.

### Next-generation sequencing

DNA was extracted from fresh bone marrow aspirates and next-generation sequencing (NGS) testing was performed using a targeted next-generation sequencing (NGS) panel that included 42 genes commonly mutated in myeloid neoplasms: *ANKRD26, ASXL1, BCOR, CALR, CBL, CEBPA, CSF3R, DDX41, DNMT3A, ELANE, ETNK1, ETV6, EZH2, FLT3, GATA1, GATA2, IDH1, IDH2, JAK2, KDM6A, KIT, KRAS, MPL, NPM1, NRAS, PHF6, PTPN11, RAD21, RUNX1, SETBP1, SH2B3, SF3B1, SRP72, SMC3, SRSF2, STAG2,TERT, TET2, TP53, U2AF1, WT1*, and *ZRSR2*. The library preparation, sequencing, and data analysis were performed as described [15]. Briefly, libraries were prepared using the Agilent SureSelect‐XT Target Enrichment Kit (SureSelectXT, Agilent, Santa Clara, CA). and sequencing was performed on MiSeq or HiSeq platforms (Illumina, San Diego, CA) at the Mayo Clinic Clinical Genome Sequencing Laboratory. Pathogenic and likely pathogenic variants calling was performed as described [16]. Only the variants at the sites with a total read depth >100, supported by more than five alternate variant reads and a variant allele frequency (VAF) ≥ 5%, were retained for further analysis.

### Statistical analysis

Univariate analysis was performed using logistic regression for nominal characteristics and Cox proportional hazard for time-to-event endpoints. Myeloid neoplasm-free survival (MNFS) was defined as interval from t-CC diagnosis to t-MN progression or the last follow-up. Progression-free survival (PFS) was defined as interval from t-CC diagnosis to t-MN progression or death. Finally, overall survival (OS) was calculated from t-CC diagnosis to last follow-up or death, whichever occurred first. Kruskal–Wallis test for continuous variables and Fisher Exact test for categorical variables were used with a significance level of 5% or less (*P*-value ≤0.05). Statistical analysis was performed using BlueSky Statistics (Chicago, IL) and figures were generated using GraphPad (v9, San Diego, USA). Oncoplot was prepared as described [17, 18].

## Results

### Clinical and pathological characteristics

**Fig. 1:**
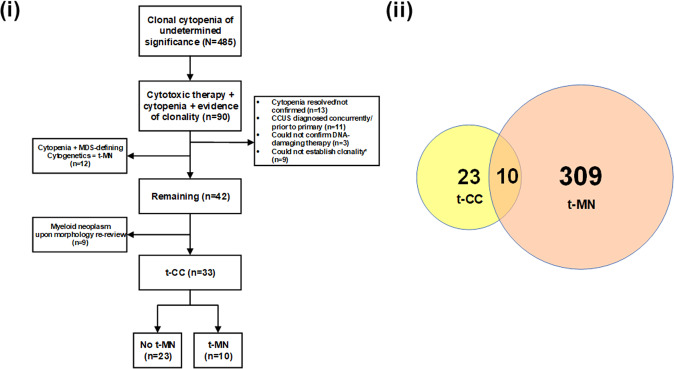
Experimental design and description of the cohort. (i) CONSORT diagram; and (ii) Venn diagram showing the relation between therapy-related clonal cytopenia (t-CC) and therapy-related myeloid neoplasm (t-MN) patients. *1 patient did not have NGS analysis performed. CCUS clonal cytopenia of undetermined significance. MDS myelodysplastic syndrome.

**Fig. 2:**
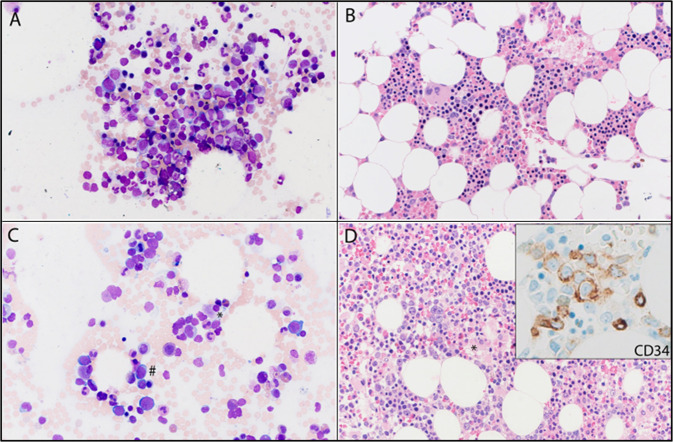
A representative example of the morphological changes associated with progression from therapy-related clonal cytopenia (t-CC) to therapy-related myeloid neoplasm (t-MN). **A** Bone marrow aspirate smear (600x) and **B** bone marrow biopsy (400x) at the time of t-CC diagnosis. Six months later, **C** bone marrow aspirate smear (600x) and **D** bone marrow biopsy (400x) showed dysplastic changes in the megakaryocytes (*) and increase in CD34+ myeloblasts (# and inset), consistent with the diagnosis of t-MN.

We identified 90 patients who developed unexplained cytopenia following cytotoxic therapy. Of these, 36 were excluded for reasons as shown (Fig. 1). Twelve patients with no morphologic evidence of a myeloid neoplasm but the presence of MDS-defining cytogenetic abnormalities [referred to as t-MDS (cyto)] were classified as t-MN according to the WHO guideline [13]. The remaining 42 cases were re-reviewed, of which 9 (21.4%) cases were determined to have t-MN, while 33 patients were determined to have t-CC. Detailed pathological features of t-CC patients are described in Table 1 and serial bone marrow examinations from a representative case are shown (Fig. 2). We also identified 309 WHO-defined t-MN patients and compared clinicopathological features of the two cohorts (Supplementary Table 1). The interval from the primary diagnosis to t-CC was shorter compared to t-MN (34.4 vs. 79.8 months, *P* < 0.001). t-CC patients were more likely to have received IST (30.3% vs. 8.7%, *P* = 0.001); whereas a higher proportion of t-MN patients had received chemotherapy (82.5% vs. 63.6%, *P* = 0.018). Presentation as t-MN was associated with a higher degree of anemia (9 vs. 10.9 g/dL, *P* < 0.001), absolute neutropenia (1.1 vs. 1.6, *P* = 0.022), and thrombocytopenia (63 vs. 101, *P* = 0.037); though the white blood cell (WBC) count did not differ between the two cohorts. t-CC patients had a lower likelihood of abnormal cytogenetics (24.2% vs. 84.9%, *P* < 0.001), complex karyotype (CK, none vs. 52.3%, *P* < 0.001), and monosomal karyotype (MK, none vs. 50.3%, *P* < 0.001) compared to t-MN. Finally, we compared the clinicopathological features of t-CC, t-MDS (cyto), and t-MN separately and found that t-MDS (cyto) had distinct clinicopathological features (Supplementary Table 2 and Supplementary Fig. 1).

**Table 1:** Pathological features at the time of therapy-related clonal cytopenia (t-CC).

UPIN	Age at t-CC	Gender	Cytopenia			Cellularity	M:E ratio	Precursor quantity			Evidence of dysplasia?^a^			Myeloid MFC	Final phenotype
			Hgb	WBC	Plts.			Normo.	Myeloid	Meg.	Erythroid	Myeloid	Meg.		
1012	60	M	Y	N	Y	40	1.3	↑	↓	↑	Subtle	No	Subtle	Normal	t-MN
1035	55	F	N	Y	N	70	4	Normal	Normal	↑	No	No	Subtle	Normal	No t-MN
1045	77	M	Y	N	N	80		↑	↑	↑	No	Subtle	Subtle	Normal	No t-MN
1067	57	F	Y	Y	Y	50	1.5	Normal	Normal	Normal	Normal	No, toxic changes^b^	Normal	Abnormal	t-MN
1132	57	M	Y	N	Y	40	2	Normal	Normal	Normal	Subtle	No	No	Normal	t-MN
1142	45	F	N	N	Y	50	2	Normal	↑	↑	No	No	No		t-MN
1151	77	M	Y	N	N	45	2	Normal	Normal	Normal	Subtle	Subtle	Subtle	Abnormal	No t-MN
1178	78	M	Y	N	Y	20	3	Normal	Normal	Normal	No	No	Subtle	Normal	No t-MN
1193	69	M	Y	Y	N	70	8	↓	↑	↑	No	No	No	Normal	No t-MN
1204	77	M	N	Y	N	35	4	↑	↑	↑	No	Subtle	Subtle	Abnormal	No t-MN
1247	59	M	N	N	Y	90	3	↑	↑	Normal	Subtle	Subtle	No	Abnormal	No t-MN
1251	67	F	Y	Y	Y	10	3	↓	↓	↓	No	No	No		No t-MN
1255	66	M	Y	Y	Y	35	1	↑	↑	Normal	No	No	No	Normal	No t-MN
1257	50	F	N	Y	N	50	4	Normal	Normal	Normal	No	No	No	Normal	No t-MN
1266	67	F	Y	Y	N	35	3	Normal	Normal	Normal	No	No	No		No t-MN
1268	58	F	N	Y	N	50	3	Normal	Normal	Normal	No	No	No	Normal	No t-MN
1275	67	M	Y	N	N	15	4	Normal	Normal	Normal	No	No	No	Normal	No t-MN
1277	68	F	N	Y	N	30	2	Normal	Normal	Normal	Subtle	No	No	Normal	No t-MN
1724	51	F	Y	N	Y	65	2.3	Normal	↑	↑	Subtle	Normal	Subtle		t-MN
2004	72	M	Y	Y	Y	30	1	↑	↓	Normal	No	No	No	Normal	t-MN
2040	67	F	Y	Y	Y	50	1.5	Normal	Normal	Normal	No	No	Subtle	Normal	No t-MN
2042	77	M	Y	N	Y	70	2	↑	↑	↑	Subtle	No	No	Normal	No t-MN
2043	66	M	Y	N	Y	40	6	↓	↑	↓	No	Subtle	No		t-MN
2049	68	F	Y	N	N	20	0.5	↑	↓	↓	Subtle	No	No	Abnormal	No t-MN
2050	74	M	N	N	Y	50	3	↑	↑	Normal	No	No	Subtle	Normal	No t-MN
2052^c^	71	M	Y	N	Y	50	1.4	Normal	Normal	Normal	No	No	No	Normal	t-MN
2053	65	M	Y	N	Y	30	1	↑	↓	Normal	No	No	No		No t-MN
2054	62	F	N	N	Y		1		Normal	Normal	Subtle	No	No	Abnormal	No t-MN
2056	69	M	Yes	No	Yes	30	0.6	↑	↓	↓	Subtle	Normal	Normal		t-MN
2057	61	F	Yes	Yes	Yes	20		↓	↓	↓	Normal	Normal	Normal		t-MN
2077	70	M	Yes	Yes	Yes	20	1.1	↓	↓	↓	Normal	Normal	Normal		No t-MN
2078	83	F	No	Yes	No	20	0.3	↑	↓	Normal	Subtle	Subtle	Normal		No t-MN
2079	78	M	No	No	Yes	60	4.0	Normal	↑	Normal	Normal	Subtle	Normal		No t-MN

*UPIN* unique patient identification number, *Hgb* hemoglobin, *WBC* white blood count, *Plts* platelets, *cellularity* percent bone marrow cellularity, *M:E* myeloid to erythroid ratio, *Normo.* normoblasts, *Meg.* megakaryocytes, *MFC* multiparametric flow cytometry, *t-MN* therapy-related myeloid neoplasm. ↑ - increased, ↓ - decreased.

^a^Morphological findings were nondiagnostic of myeloid neoplasm in all cases.

^b^Left-shifted maturation with toxic changes in the setting of an intraabdominal infection and recent G-CSF.

^c^NGS performed using peripheral blood.

**Fig. 3:**
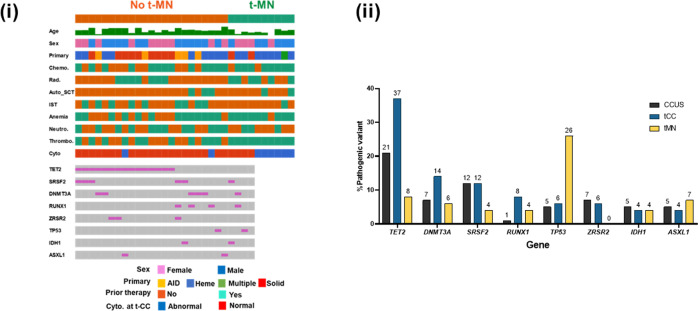
Comparing genetic landscapes of therapy-related clonal cytopenia (t-CC), clonal cytopenia of undetermined significance (CCUS), and therapy-related myeloid neoplasms (t-MN). (i) Clinical and genetic characteristics of t-CC patients; and (ii) the distribution of pathogenic variants in clonal cytopenia of undetermined significance (CCUS), therapy-related clonal cytopenia (t-CC), and therapy-related myeloid neoplasm (t-MN). AID autoimmune disease, Heme primary hematological malignancy, Solid primary solid tumor, Chemo chemotherapy, Rad radiation, Auto SCT autologous stem cell transplant, IST immunosuppressive therapy, Neutro neutropenia, Thrombo thrombocytopenia, cyto cytogenetics.

The proportion of patients with abnormal NGS was similar between t-CC and t-MN (96.3% vs. 90.3%, *P* = 0.477). Clinicopathological features and its correlation with NGS findings are shown in Fig. 3 and Supplementary Table 3. Median variance allele frequency (VAF) at t-CCUS was 35% (range 5–76%). The median VAF was not different between the *DTA* and non-*DTA* genes (33% vs. 39%, *P* = 0.45). The proportion of patients with a pathogenic variant (PV) in *TP53* was significantly higher in t-MN compared to t-CC (40.9% vs. 7.4%, *P* < 0.001). On the other hand, a higher proportion of t-CC patients had PV in *TET2* (55.6% vs. 10%, *P* < 0.001), and *SRSF2* (23.1% vs. 8.1%, *P* = 0.028) compared to t-MN. The most common PV in t-CC were in *TET2* 19 (37%, Fig. 3), *DNMT3A* 7(14%), *SRSF2* 66(12%), *RUNX1* 4 (8%), and *TP53* 3 (6%). In contrast, the most common PVs in CCUS [9] were *TET2* (21%), *SRSF2* (12%), *DNMT3A* (7%), *ZRSR2* (7%), and *U2AF1* (6%) and the most common PV in t-MN were *TP53* (26%), *TET2* (8%), *ASXL1* (7%), *DNMT3A* (6%), *SRSF2* (4%) and *IDH1* (4%). Thus, the genetic landscape of t-CC is distinct from t-MN as well as compared to the recently described cohort of CCUS patients [9].

### Risk of t-MN progression

At the last follow-up, 10 (30.3%) patients progressed to t-MN. We compared the characteristics of t-CC patients that subsequently developed t-MN compared to those who did not (Table 2). The distribution of the primary condition was different between the two cohorts: a higher proportion of patients that developed t-MN had hematological malignancy, whereas none of the 5 patients with AID developed t-MN. A higher degree of thrombocytopenia (platelets 46 vs. 11, *P* = 0.012), the presence of bone marrow blasts (1 vs. 0, *P* = 0.021), and nucleated red blood cells (0.6 vs. 0, *P* < 0.001) were associated with a higher likelihood of a subsequent t-MN. Similarly, the presence of a cytogenetic abnormalities (*P* = 0.004) and the absence of *DTA* variants (*P* = 0.015) were associated with a higher likelihood of a subsequent t-MN. Regardless of the presence of a non-*DTA* variants, the presence of at least one *DTA* variant was associated with a low risk of a subsequent t-MN: none of the 12 patients with *DTA* variants only developed t-MN, whereas 1 of the 8 patients with combined *DTA* and non-*DTA* variants progressed to t-MN.

**Table 2:** Clinicopathological characteristics therapy-related clonal cytopenia (t-CC) patients that progressed to therapy-related myeloid neoplasm (t-MN) and those who do not.

Variables	No subsequent t-MN (*N* = 23)	Subsequent t-MN (*N* = 10)	*P*-value
Hemoglobin g/dL, median (Q1, Q3)	11.2 (8.9, 12.6)	10.2 (7.8, 12.3)	0.372
Mean corpuscular volume, median (Q1, Q3)	99.8 (94.8, 109.6)	105.3 (102.2, 108.3)	0.412
Red cell distribution width, median (Q1, Q3)	14.2 (13.7, 17.2)	15.2 (15.2, 17.2)	0.218
White blood cell, median (Q1, Q3)	3.2 (2.1, 4.4)	3.8 (2.1, 5.2)	0.811
Platelets, median (Q1, Q3)	**110.0 (78.2, 166.2)**	**46.0 (34.0, 80.0)**	**0.012**
Absolute neutrophil count, median (Q1, Q3)	1.1 (0.8, 2.4)	1.8 (1.2, 6.3)	0.361
% Blasts, median (Q1, Q3)	**0.0 (0.0, 0.0)**	﻿**1.0 (0.0, 2.5)**	**0.021**
% Nucleated red cells, median (Q1, Q3)	**0.0 (0.0, 0.0)**	**0.6 (0.0, 1.0)**	**<0.001**
% Cellularity, median (Q1, Q3)	40.0 (22.5, 57.5)	40.0 (30.0, 42.5)	0.777
M:E ratio, median (Q1, Q3)	3.0 (1.1, 4.0)	2.0 (1.1, 2.1)	0.362
Gender			1
Female	10 (43.5%)	4 (40.0%)	
Male	13 (56.5%)	6 (60.0%)	
Primary condition			0.069
Hematological malignancy	8 (36.4%)	7 (70.0%)	
Solid malignancy	9 (40.9%)	2 (20.0%)	
Multiple malignancies	0	1 (10%)	
Autoimmune disease	5 (22.7%)	0	
Chemotherapy			0.259
No	10 (43.5%)	2 (20.0%)	
Yes	13 (56.5%)	8 (80.0%)	
Radiation			0.139
No	16 (69.6%)	4 (40.0%)	
Yes	7 (30.4%)	6 (60.0%)	
Prior autologous stem cell transplant			0.627
No	20 (87.0%)	8 (80.0%)	
Yes	3 (13.0%)	2 (20.0%)	
Immunosuppressive therapy			0.123
No	14 (60.9%)	9 (90.0%)	
Yes	9 (39.1%)	1 (10.0%)	
Anemia at t-CC diagnosis			0.109
No	10 (43.5%)	1 (10.0%)	
Yes	13 (56.5%)	9 (90.0%)	
Neutropenia at t-CC diagnosis			0.283
No	11 (47.8%)	7 (70.0%)	
Yes	12 (52.2%)	3 (30.0%)	
Thrombocytopenia at t-CC diagnosis			**0.005**
No	**12 (52.2%)**	**0 (0.0%)**	
Yes	**11 (47.8%)**	**10 (100.0%)**	
Cytogenetics at t-CC diagnosis			**0.004**
Abnormal	**2 (8.7%)**	**6 (60.0%)**	
Normal	**21 (91.3%)**	**4 (40.0%)**	
NGS at t-CC diagnosis			0.179
Abnormal	23 (100.0%)	4 (80.0%)	
Normal	0 (0.0%)	1 (20.0%)	
PV in *DTA* genes			**0.015**
Absent	**19 (82.6%)**	**1 (20.0%)**	
Present	**4 (17.4%)**	**4 (80.0%)**	
PV in *TP53*			0.279
No	22 (95.7%)	3 (75.0%)	
Yes	1 (4.3%)	1 (25.0%)	
PV in *TET2*			**0.028**
No	**8 (34.8%)**	**4 (100.0%)**	
Yes	**15 (65.2%)**	**0 (0.0%)**	
PV in *DNMT3A*			1
No	18 (78.3%)	3 (75.0%)	
Yes	5 (21.7%)	1 (25.0%)	
PV in *ASXL1*			1
No	21 (91.3%)	4 (100.0%)	
Yes	2 (8.7%)	0 (0.0%)	
PV in *RAS*			1
No	22 (95.7%)	4 (100.0%)	
Yes	1 (4.3%)	0 (0.0%)	

*t-MN* therapy-related myeloid neoplasm, *M:E* myeloid to erythroid ratio, *t-CC* therapy-related clonal cytopenia, *NGS* next-generation sequencing, *PV* pathogenic variant. Fisher’s Exact test was used for categorical variables and Kruskal–Wallis was used for continuous variables.

Bold values indicates statistical significant *P* values (*P* ≤ 0.05).

**Fig. 4:**
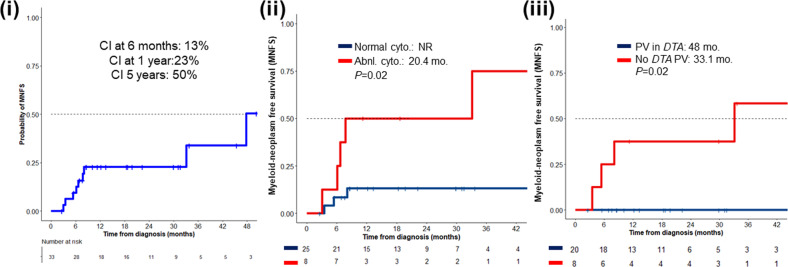
Cumulative incidence of therapy-related myeloid neoplasms (t-MN) in patients with therapy-related clonal cytopenia (t-CC). (i) Cumulative incidence (CI) of t-MN in patients with therapy-related clonal cytopenia (t-CC); (ii) Myeloid neoplasm free survival (MNFS) stratified by the presence of abnormal cytogenetics and (iii) the presence of pathogenic variants in *DNTM3A*, *TET2*, or *ASXL1* (*DTA*) genes. Abnl abnormal, cyto. cytogenetics, *DTA DNMT3A*, *TET2*, and *ASXL1* genes, PV pathogenic variant, NR not reached.

The cumulative incidence of t-MN at 6 months, 1 year, and 5 years was 13%, 23%, and 50% respectively (Fig. 4). The presence of cytogenetic abnormalities at the time of t-CC diagnosis was associated with a statistically shorter MNFS (20.4 months vs. not reached, *P* = 0.02, Supplementary Table 4). Similarly, the absence of *DTA* variants was also associated with a statistically shorter MNFS (33.1 vs. 48 months, *P* = 0.02).

### Clonal evolution at the time of t-MN progression

Paired cytogenetics and NGS at t-CC and t-MN were available for 9 and 4 patients, respectively. Combined, 4 (44.4%) of 9 evaluable patients had identifiable clonal evolution (Table 3) at t-MN progression. Among those with paired cytogenetic analyses, 3 patients acquired additional cytogenetic abnormalities (deletion of chromosome 13q, del 7q/trisomy 21, and a complex clone). Among those with paired NGS, one patient each noted the acquisition of *NRAS* and *CEBPA/TP53*, whereas in one patient each, *DNMT3A*/*RUNX1* and *TP53* were undetectable. Thus, leukemic transformation was associated with diverse biological mechanisms.

**Table 3:** Clonal evolution at the time of progression to therapy-related myeloid neoplasm.

UPIN	t-CC to t-MN*	Cytogenetics at t-CC	# PV at t-CC	PV at t-CC (%VAF)	Cytogenetics at t-MN	# PV at t-MN	PV at t-MN (%VAF)	Summary
1012	6.6	46,XY,del(7)(p15)[20]	Not performed		46,XY,del(7)(p15)[20]	0	None	No change
1067	3.5	46, XX	2	*BCOR (*23%*), U2AF1 (*26%*)*	46, XX	3	*BCOR (36%), U2AF1 (45%), NRAS (43%)*	Clonal evolution
1132	3.0	47,XY, + 21[3]/46, XY[17]	Not performed		47,XY, + 21[7]/46,XY[13].	2	*SRSF2* (44%), *U2AF1* (36%)	No change
1142	98.7	Trisomy 8	Not performed		47,XX, + 8[6]/46,XX[14]	Not performed		No change
1724	47.8	46, XY	2	*DNMT3A* (8%), *RUNX1* (48%)	46,XX,del(13)(q12q14)[7]/46,XX[13]	0	None	Clonal evolution
2004	33.1	46,XY,del(4)(q21q31),t(7;20)(q22;q13.1)[4]/ 46,XY[16].	0	None	45,XY,del(5)(q22q31),add(7)(q22),-14,add(18)(q21), add(19)(p13.1)[18]/ 45,XY,del(5)(q22q31),add(6)(p23),-18[2].	2	*TP53 (25%) CEBPA (7%)*	Clonal evolution
2043	6.1	47,XY, + 8[11]/46,XY[9]	Not performed		47,XY, + 8[8]/46,XY[12]	6	*ASXL1* (46%), *BCOR* (65%), *EZH2* (44%) *EZH2* (50%), *RUNX1* (13%), *TET2* (44%)	No change
2052	8.1	46, XY	2	*IDH1* (33%), *SRSF2* (27%)	46, XY	Not performed		No change
2057	5.4	46, XY	2	*TP53* (5.5%), *TP53* (7.5%)	46,XY,del(7)(q22)[11]/47,idem,+21[5]/46,XY[4]	0	None	Clonal evolution

*UPIN* unique patient identification number, *PV* pathogenic variant, *VAF* variance allele frequency, *t-CC* therapy-related clonal cytopenia, *t-MN* therapy-related myeloid neoplasm.

*interval in months.

### Outcomes following t-CC

Most common management strategy for t-CC was observation in 19 patients. The rest were treated with growth factor support (n = 7), chemotherapy (n = 1), IST (n = 1), intravenous immunoglobulin (n = 1), or a clinical trial (n = 1). One patient was diagnosed as MDS at an outside institution and underwent allogeneic SCT. At the last follow up, 11 (33.3%) deaths were noted. Primary causes of death included: t-MN (n = 4), infection (n = 2), cardiac complications (n = 2), primary malignancy (n = 1), progressive cytopenia without MN (n = 1), and undetermined (n = 1). Thus, 4 of 11 deaths were noted in patients who did not develop a subsequent t-MN.

**Fig. 5:**
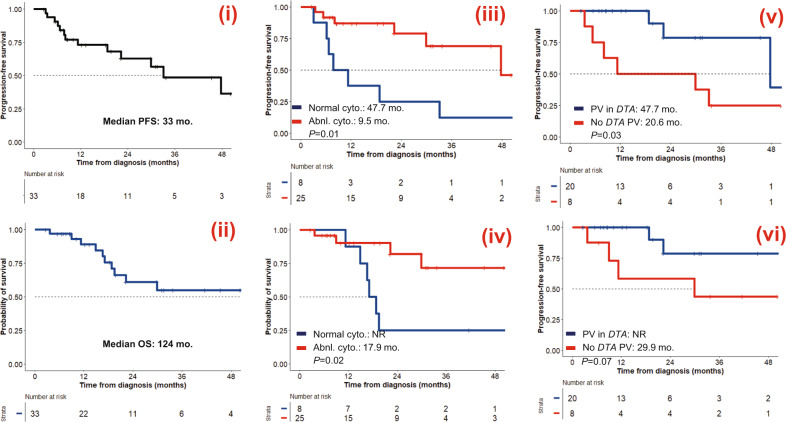
Outcomes following the development of therapy-related clonal cytopenia (t-CC). (i) Progression-free survival (PFS), and (ii) overall survival (OS) of the entire AQ11 cohort. (iii) PFS and (iv) OS stratified by the presence of abnormal cytogenetics.(v) PFS and OS (vi) stratified by the presence of pathogenic variants in *DNTM3A*, *TET2*, or *ASXL1* (*DTA*) genes. Abnl abnormal, cyto. cytogenetics, *DTA DNMT3A*, *TET2*, and *ASXL1* genes, PV pathogenic variant, NR not reached.

Median PFS and OS for the entire cohort were 33 and 124.5 months respectively (Fig. 5). The presence of cytogenetic abnormality (9.5 vs. 47.7 months, *P* = 0.01) and the absence of *DTA* variants (20.6 vs. 47.7 months, *P* = 0.03) were associated with a shorter PFS. Similarly, the presence of cytogenetic abnormality (17.9 months vs. not reached, *P* = 0.02) was associated with inferior OS and there was a trend towards an inferior survival in the absence of *DTA* variants (29.9 months vs. not reached, *P* = 0.07). Other factors associated with shorter PFS were the history of autologous SCT (HR 5.02, *P* = 0.03, Supplementary Table 5), the presence of thrombocytopenia (HR 9.1, *P* = 0.03), or the presence of anemia (HR 8.58, *P* = 0.04) at t-CC diagnosis. Similarly, history of autologous stem cell transplant (HR 8.82, *P* = 0.01, Supplementary Table 6) was associated with an inferior OS.

### Outcomes following t-CC compared to t-MN

**Fig. 6:**
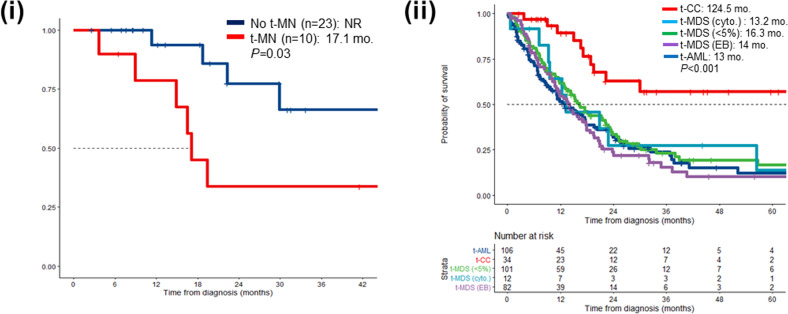
Therapy-related clonal cytopenia (t-CC) is a distinct clinical entity characterized by superior survival compared to therapy-related myeloid neoplams (t-MN). (i) Overall survival (OS) in therapy-related clonal cytopenia (t-CC) as stratified by the subsequent development of therapy-related myeloid neoplasm (t-MN); (ii) t-CC has significantly superior OS compared to t-MN, including compared to t-MDS (<5% bone marrow blasts). t-MDS (cyto)—therapy-related myelodysplastic syndrome based on the presence of MDS-defining cytogenetics; t-MDS (<5%)—t-MDS with <5% blasts at the time of presentation; t-MDS (EB)—t-MDS with excess blasts (5–19%); t-AML—therapy-related acute myeloid leukemia.

We next assessed the impact of the development of a subsequent t-MN on survival. Patients who developed t-MN (*n* = 10) had a statistically significantly inferior survival compared to those who did not (17.1 months vs. not reached, *P* = 0.03, Fig. 6). Comparing the overall survival following t-CC to that with various phenotypic subclassifications of t-MN: t-MDS with <5% bone marrow blasts, t-MDS with excess blasts (t-MDS-EB), t-MDS/MPN, and t-AML showed that t-CC patients had a significantly superior survival (124.5 months) compared to t-MDS with <5% blasts (16.3 months), t-MDS-EB (14 months), and t-AML (13 months).

### The significance of MDS-defining cytogenetic abnormalities

Of the 12 t-MDS (cyto) patients, 9 (75%) developed the morphological evidence of a myeloid neoplasm at a median of 10.5 months, while 3 (25%) patients did not. One patient (UPIN 2048) died within 1 month due to sepsis. UPIN 2041 died of progressive lymphoma 12 months without developing morphological evidence of a myeloid neoplasm. Finally, UPIN 1036 was alive at 44 months despite harboring the deletion of chromosome 5q, as well as PVs in *ASXL1* and *TP53*. Time to develop morphological evidence of t-MN was significantly shorter for those with t-MDS(cyto) compared to t-CC (Supplementary Table 7, Supplementary Fig. 2). Importantly, median OS of the patients with MDS-defining cytogenetic abnormalities was significantly inferior compared to t-CC patients (13.2 vs. 124 months, *P* = 0.01), but was comparable to the other t-MN phenotypes (13–16.3 months, Fig. 5).

## Discussion

Therapy-related myeloid neoplasms are one of the most aggressive malignancies with no effective therapies and an exceedingly poor survival [20]. Therefore, there is an urgent need to predict and prevent future t-MN.

The evidence of clonality in patients with otherwise unexplained cytopenia, impart a 17–27% risk of developing a subsequent myeloid neoplasm [9–11]. However, these studies did not assess the impact of prior DNA-damaging therapies, which is one of the strongest known risk factors for myeloid neoplasms [21].

We comprehensively characterized CCUS developing after the exposure to DNA-damaging therapies and found that the cumulative incidence of t-MN was 50% at 5 years. The interval from the diagnosis of primary malignancy/AID to t-CC diagnosis was shorter than t-MN. In addition, t-MN was accompanied by a higher degree of anemia, absolute neutropenia, and thrombocytopenia. A significantly smaller proportion of t-CC patients had *TP53*, CK, and MK—features known to predict inferior outcomes [3]. This raises two possibilities—the first is that patients presenting as t-CC, including those progressed to t-MN, represent a distinct subset of t-MN. Another possibility is that the leukemic transformation is a distinct biological event characterized by the acquisition of high-risk features including the acquisition of PV in *TP53* as well as chromosomal instability. While limited by a small cohort size, the paired cytogenetic/genetic analysis at t-CC and t-MN showed complex clonal evolution seen at the time of leukemic transformation. Using a lower VAF threshold (≥2%), a recent study found that the leukemic transformation was associated with acquisition of additional somatic mutations, including chromosomal aneuploidies or mutations in genes in 91% of cases [8]. Combined, these observations raise the possibility that a prompt and accurate diagnosis of t-CC may allow for interventions which may not be feasible in t-MN patients.

Designing optimized surveillance strategies and counseling will require an accurate identification of patients at a higher risk of t-MN. We noted that the presence of cytogenetic abnormalities was associated with inferior outcomes, whereas the presence of *DTA* variants—regardless of the co-existing non-*DTA* variants—were associated with superior outcomes. The mechanism underlying this observation remains unclear. A possible explanation is that the presence of *DTA* variants may denote ‘true’ CHIP-like clone, whereas the presence of cytogenetic abnormalities and/or non-*DTA* variants may represent a therapy-related clone.

Long-term follow up following t-CC revealed 2 interesting themes: more than a third of patients died without developing t-MN. These findings are commensurate with CHIP patients who experience increased morbidity and mortality [1, 19]. On the other hand, the survival of t-CC patients was significantly superior compared to t-MN patients. In the de novo context, an argument can be made that the clear distinction between CCUS and low-risk MDS (LR-MDS) may be of little importance; as observation is the preferred option for both entities [1], and there is no difference in survival [9]. This is in stark contrast with our findings as t-CC appears to have superior survival compared to all t-MN phenotypes, including t-MDS with no increase in blasts. In addition, once diagnosed with t-MN, very few patients would be watched without interventions. Thus, we argue that the distinction between t-CC and t-MN is clinically meaningful.

Finally, we assessed if, in the context of prior DNA-damaging therapy, the presence of MDS-defining cytogenetic abnormalities carries similar prognostic significance as in the de novo context [12, 13]. Survival of t-MDS(cyto) patients was no different compared to t-MN, but significantly superior to t-CC—supporting the assertion that even in the absence of morphological evidence of a myeloid neoplasm, these cases should be considered as t-MN. However, the interval from the emergence of cytogenetic abnormality to morphological progression varied greatly (0.9–81 months) and 2 patients did not develop morphological evidence of t-MN despite 12 and 44 months of follow-up. Goswami et al. followed patients with prior DNA-damaging therapy who developed isolated deletion of 7q—an MDS-defining cytogenetic anomaly [12]. While these patients fulfilled the WHO-defined t-MN diagnostic criteria, less than half actually progressed to t-MN. Collectively, these results underline the heterogeneity of the cohort and the need for larger studies that will help more accurate risk stratification of this cohort.

A limitation of our study was that the samples obtained prior to DNA-damaging therapy were not available and that a subset of patients did not have paired NGS performed at t-CC and t-MN. Therefore, whether a clone strictly represented CHIP, or the effect of the prior DNA-damaging therapy could not be established. Second, the absolute and relative risks of t-CC or t-MN following chemotherapy could not be inferred. Third, *PPM1D* that has a well-known association with t-MN; [22] as well as *CUX1* [23], recently described to be a gatekeeper in t-MN pathogenesis, were not assessed. Fourth, with regards to the paired sequencing, the inability to identify a corresponding t-MN clone at the time of t-CC does not necessarily denote the absence of such a clone. It is possible that low-level clone is present below the detection threshold of NGS and cytogenetics technique. Finally, given that t-CC, at least in some cases, acted as a precursor to t-MN, whether the difference in outcomes reflects the differences in the biology of the 2 entities or the lead-time bias could not be ascertained [24]. Therefore, a larger prospective study of all patients undergoing cytotoxic therapies with a longer follow-up will be needed to answer these questions.

In summary, we describe t-CC as a putative precursor entity of t-MN and identify the risk factors for poor outcomes including the progression to t-MN. t-CC had a distinct clinical and genetic profile as well as overall superior survival compared to t-MN. The presence of cytogenetic abnormality and the absence of variants in *DNMT3A*, *TET2*, or *ASXL1* genes were associated with a higher likelihood of progressing to t-MN and an inferior survival. Paired analysis at t-CC and t-MN as well as comparative analyses of the t-CC and t-MN cohorts suggest that the leukemic transformation as an event characterized by acquisition of morphologic evidence of dysplasia as well as cytogenetic and genetic abnormalities. Given that half of the patients develop t-MN over the next 5 years, a wider recognition of t-CC may allow to individualize counseling, optimize surveillance, and design prevention studies—ultimately improving outcomes in t-MN.

## Supplementary information

Author Checklist

Supplementary Material

## Data Availability

All data generated or analyzed during this study are included in this published article (and its supplementary information files).
