# Supplementary material for: Therapy-related clonal cytopenia as a precursor to therapy-related myeloid neoplasms
Source: Blood Cancer J. 2022 Jul 8;12(7):106. doi: 10.1038/s41408-022-00703-8 (PMC9270475; doi:10.1038/s41408-022-00703-8)
Supplement: Supplementary file 2 — Supplementary Material [file 41408_2022_703_MOESM2_ESM.pdf]

Supplementary Material

Shah *et al*. Therapy-related clonal cytopenia as a precursor to therapy-related myeloid neoplasms

(A) Supplementary Tables

Supplementary Table 1: Clinical and pathological characteristics of therapy-related clonal cytopenia (t-CC) and therapy-related myeloid neoplasms (t-MN)

Supplementary Table 2: Clinical and pathological characteristics of patients with therapy-related clonal cytopenia (t-CC), MDS-defining cytogenetic abnormalities (t-MDS cyto), and therapy-related myeloid neoplasms (t-MN)

Supplementary Table 3: Variants identified at the time of the diagnosis of therapy-related clonal cytopenia (t-CC)

Supplementary Table 4: Cox proportional hazard analysis for myeloid neoplasm free survival (MNFS)

Supplementary Table 5: Cox proportional hazard analysis for progression free survival (PFS)

Supplementary Table 6: Cox proportional hazard model for overall survival (OS)

Supplementary Table 7: Outcomes of patients with clonal cytopenia and the presence of myelodysplastic syndrome (MDS)-defining cytogenetics

Supplementary Table 1: Clinical and pathological characteristics of t-CC and t-MN

Variable	t-CC* (N=33)	t-MN (N=309)	*P*-value
Age at primary, median (Q1, Q3)	63.9 (57.5, 67.1)	59.3 (51.2, 66.0)	0.055
Age at t-CC or t-MN diagnosis, median (Q1, Q3)	67.3 (60.3, 72.2)	67.9 (60.5, 73.7)	0.744
**Primary to t-CC or t-MN in months, median (Q1, Q3)**	**34.4 (17.4, 80.1)**	**79.8 (45.0, 145.9)**	**< 0.001**
Gender			1.000
- Female	14 (42.4%)	135 (43.7%)	
- Male	19 (57.6%)	174 (56.3%)	
Chemotherapy			0.018
- No	12 (36.4%)	54 (17.5%)	
- Yes	21 (63.6%)	255 (82.5%)	
Radiation			0.364
- No	20 (60.6%)	160 (51.8%)	
- Yes	13 (39.4%)	149 (48.2%)	
Autologous stem cell transplantation			0.504
- No	28 (84.8%)	240 (77.7%)	
- Yes	5 (15.2%)	69 (22.3%)	
Immunosuppressive therapy			0.001
- No	23 (69.7%)	282 (91.3%)	
- Yes	10 (30.3%)	27 (8.7%)	
Hemoglobin (g/dL), median (Q1, Q3)	10.9 (8.6, 12.5)	9.0 (7.8, 10.8)	0.001
White blood count, median (Q1, Q3)	3.6 (2.1, 4.8)	2.8 (1.7, 5.4)	0.518
Platelets, median (Q1, Q3)	101.0 (48.5, 139.5)	63.0 (33.0, 107.5)	0.037
Absolute neutrophil count, median (Q1, Q3)	1.6 (0.9, 2.1)	1.1 (0.4, 1.9)	0.022
Cytogenetics at diagnosis			< 0.001
- Abnormal	8 (24.2%)	259 (84.9%)	
- Normal	25 (75.8%)	46 (15.1%)	
- Not available	0	4	
Complex karyotype			< 0.001
- No	33 (100.0%)	145 (47.7%)	
- Yes	0 (0.0%)	159 (52.3%)	
- Not available	0	5	
Monosomal karyotype			< 0.001
- No	33 (100.0%)	151 (49.7%)	
- Yes	0 (0.0%)	153 (50.3%)	
- Not available	0	5	
NGS at diagnosis			0.477
- Abnormal	26 (96.3%)	159 (90.3%)	
- Normal	1 (3.7%)	17 (9.7%)	
- Not available	6	133	
PV in *TP53*			< 0.001
- No	25 (92.6%)	120 (59.1%)	
- Yes	2 (7.4%)	83 (40.9%)	
- Not available	6	106	
PV in *TET2*			< 0.001
- No	12 (44.4%)	180 (90.0%)	
- Yes	15 (55.6%)	20 (10.0%)	
- Not available	6	109	
PV in *DNMT3A*			0.14
- No	21 (77.8%)	176 (88.0%)	
- Yes	6 (22.2%)	24 (12.0%)	
- Not available	6	109	
PV in *ASXL1*			0.748
- No	25 (92.6%)	176 (88.0%)	
- Yes	2 (7.4%)	24 (12.0%)	
- Not available	6	109	
PV in *RAS*			0.482
- No	26 (96.3%)	180 (90.0%)	
- Yes	1 (3.7%)	20 (10.0%)	
- Not available	6	109	
PV in *SRSF2*			0.028
- No	20 (76.9%)	181 (91.9%)	
- Yes	6 (23.1%)	16 (8.1%)	
- Not available	7	112	
PV in *IDH*			1
- No	24 (92.3%)	182 (91.9%)	
- Yes	2 (7.7%)	15 (7.6%)	
- Not available	7	112	

t-CC – therapy-related clonal cytopenia; t-MN – therapy-related myeloid neoplasm; NGS – next generation sequencing; PV – pathogenic variant
*patients diagnosed with t-CC are included in the t-CC cohort even if they subsequently developed t-MN.

Supplementary Table 2: Clinical and pathological characteristics of t-CC, t-MDS (cyto), and t-MN				
**Variable**	**t-CC*<br>(N=33)**	**t-MDS (cyto)<br>(N=12)**	**t-MN<br>(N=297)**	***P*-value**
Age at primary, median (Q1, Q3)	63.9 (57.5, 67.1)	63.9 (58.9, 68.0)	59.1 (50.8, 65.8)	0.017
Age at t-CC or t-MN diagnosis, median (Q1, Q3)	67.3 (60.3, 72.2)	73.4 (65.7, 79.1)	67.5 (60.0, 73.7)	0.108
Primary to t-CC or t-MN in months, median (Q1, Q3)	34.4 (17.4, 80.1)	127.3 (64.9-209.3)	77.2 (44.9, 144.6)	<0.001
Gender<br>- Female<br>- Male	 14 (42.4%)<br>19 (57.6%)	 9 (75.0%)<br>3 (25.0%)	 126 (42.4%)<br>171 (57.6%)	0.084
Chemotherapy<br>- No<br>- Yes	 12 (36.4%)<br>21 (63.6%)	 2 (16.7%)<br>10 (83.3%)	 52 (17.5%)<br>245 (82.5%)	0.033
Radiation<br>- No<br>- Yes	 20 (60.6%)<br>13 (39.4%)	 6 (50.0%)<br>6 (50.0%)	 154 (51.9%)<br>143 (48.1%)	0.598
Autologous stem cell transplantation<br>- No<br>- Yes	 28 (84.8%)<br>5 (15.2%)	 12 (100.0%)<br>0 (0.0%)	 228 (76.8%)<br>69 (23.2%)	0.089
Immunosuppressive therapy<br>- No<br>- Yes	 23 (69.7%)<br>10 (30.3%)	 11 (91.7%)<br>1 (8.3%)	 271 (91.2%)<br>26 (8.8%)	0.001
Hemoglobin (g/dL) at presentation, median (Q1, Q3)	10.9 (8.6, 12.5)	10.5 (7.8, 12.2)	9.0 (7.8, 10.7)	0.003
White blood count at presentation, median (Q1, Q3)	3.6 (2.1, 4.8)	2.7 (2.2, 4.1)	2.8 (1.7, 5.4)	0.808
Platelets at presentation, median (Q1, Q3)	101.0 (48.5, 139.5)	70.0 (51.5, 97.0)	61.5 (32.8, 107.2)	0.11
Cytogenetics at diagnosis<br>- Abnormal<br>- Normal<br>- Not available	 8 (24.2%)<br>25 (75.8%)<br>0	 12 (100.0%)<br>0 (0.0%)<br>0	 247 (84.3%)<br>46 (15.7%)<br>4	<0.001
Complex karyotype<br>- No<br>- Yes<br>- Not available	 33 (100.0%)<br>0 (0.0%)<br>0	 5 (41.7%)<br>7 (58.3%)<br>0	 140 (47.9%)<br>152 (52.1%)<br>5	<0.001
Monosomal karyotype<br>- No<br>- Yes<br>- Not available	 33 (100.0%)<br>0 (0.0%)<br>0	 8 (66.7%)<br>4 (33.3%)<br>0	 143 (49.0%)<br>149 (51.0%)<br>5	<0.001
PV in *TP53*<br>- No<br>- Yes<br>- Not available	 25 (92.6%)<br>2 (7.4%)<br>6	 2 (22.2%)<br>7 (77.8%)<br>3	 118 (60.8%)<br>76 (39.2%)<br>103	<0.001
PV in *TET2*<br>- No<br>- Yes<br>- Not available	 12 (44.4%)<br>15 (55.6%)<br>6	 8 (88.9%)<br>1 (11.1%)<br>3	 172 (90.1%)<br>19 (9.9%)<br>106	<0.001
PV in *DNMT3A*<br>- No<br>- Yes<br>- Not available	 21 (77.8%)<br>6 (22.2%)<br>6	 8 (88.9%)<br>1 (11.1%)<br>3	 168 (88.0%)<br>23 (12.0%)<br>106	0.282
PV in *ASXL1*<br>- No<br>- Yes<br>- Not available	 25 (92.6%)<br>2 (7.4%)<br>6	 7 (77.8%)<br>2 (22.2%)<br>3	 169 (88.5%)<br>22 (11.5%)<br>106	0.456
PV in *RAS*<br>- No<br>- Yes<br>- Not available	 26 (96.3%)<br>1 (3.7%)<br>6	 9 (100.0%)<br>0 (0.0%)<br>3	 171 (89.5%)<br>20 (10.5%)<br>106	0.524
PV in *SRSF2*<br>- No<br>- Yes<br>- Not available	 20 (76.9%)<br>6 (23.1%)<br>7	 8 (88.9%)<br>1 (11.1%)<br>3	 173 (92.0%)<br>15 (8.0%)<br>109	0.034
PV in *IDH*<br>- No<br>- Yes<br>- Not available	 24 (92.3%)<br>2 (7.7%)<br>7	 8 (88.9%)<br>1 (11.1%)<br>3	 174 (92.6%)<br>14 (7.4%)<br>109	0.791

t-CC – therapy-related clonal cytopenia; t-MN – therapy-related myeloid neoplasm; NGS – next generation sequencing; PV – pathogenic variant<br>*patients diagnosed with t-CC are included in the t-CC cohort even if they subsequently developed t-MN.

**Supplementary Table 3: Variants identified at the time of the diagnosis of therapy-related clonal cytopenia (t-CC)**		
**UPIN**	**Gene**	**Variants identified (% variance allele frequency)**
2042	*ASXL1*	*ASXL1*: c.1934dup; p.Gly646Trpfs*12 (35%)
2078	*ASXL1*	*ASXL1*: c2256dup; Pro753Serfs*21 (9%)
1067	*BCOR*	*BCOR*: c.1990_2083dup; p.His695Argfs*76 (23%)
1193	*CBL*	*CBL*: c.1259G>A; p.Arg420Gln (11%)
1257	*DNMT3A*	*DNMT3A*: c.2645G>A; p.Arg882His (44%)
1275	*DNMT3A*	*DNMT3A*: c.2711C>T; p.Pro904Leu (17%)
1724	*DNMT3A*	*DNMT3A*: c.2401A>G; p.Met801Val (8%)
2050	*DNMT3A*	*DNMT3A*: c.2645G>A; p.Arg882His (39%)
2053	*DNMT3A*	*DNMT3A*: c.1174G>T; p.Glu392* (42%)
2077	*DNMT3A*	*DNMT3A*: c.2105A>T; p.Asp702Val (5%)
1193	*IDH1*	*IDH1*: c.394C>G; p.Arg132Gly (41%)
2052	*IDH1*	*IDH1*: c.395G>A; p.Arg132His (33%)
2040	*MPL*	*MPL*: c.1774C>T; p.Arg592* (13%)
2042	*NRAS*	*NRAS*: c.34G>A; p.Gly12Ser (34%)
1151	*RUNX1*	*RUNX1*: c.930del; p.Ala311Argfs*256 (44%)
1251	*RUNX1*	*RUNX1*: c.1069_1082del; p.Pro357Valfs*211 (43%)
1724	*RUNX1*	*RUNX1*:c.631G>A; p.Val211Ile (48%)
2077	*RUNX1*	*RUNX1*: c.631G>A; p.Val211Ile (48%)
1151	*SRSF2*	*SRSF2*: c.284C>T; p.Pro95Leu (44%)
1193	*SRSF2*	*SRSF2*: c.284C>G; p.Pro95Arg (43%)
2040	*SRSF2*	*SRSF2*: c.284C>A; p.Pro95His (35%)
2049	*SRSF2*	*SRSF2*: c.284C>A; p.Pro95His (39%)
2052	*SRSF2*	*SRSF2*: c.284_307del; p.Pro95_Arg102del (27%)
2079	*SRSF2*	*SRSF2*: c.284C>A; p.Pro95His (44%)
1035	*TET2*	*TET2*:c.2983A>C; p.Thr995Pro (51%)
1045	*TET2*	*TET2*: c.2177del;p.Gln726Argfs*25 (6%)
1178	*TET2*	*TET2*: c.4546C>T; p.Arg1516* (37%)
1204	*TET2*	*TET2*: c.4523_4524del; p.Ala1508Glufs*69 (22%)
1247	*TET2*	*TET2*: c.996C>A; p.Cys332* (44%)
1247	*TET2*	*TET2*: c.5189del; p.Asp1730Valfs*15 (41%)
1257	*TET2*	*TET2*: c.3270_3273del; p.Lys1090Asnfs*15 (42%)
1257	*TET2*	*TET2*:c.3954+5G>C; p.? (44%)
1266	*TET2*	*TET2*: c.3635dup; p.Leu1212Phefs*11 (14%)
1268	*TET2*	*TET2*: c.1434_1489dup, p.Thr497 (37.8%)
1268	*TET2*	*TET2*: c.3025C>T, p.Gln1009* (23.3%)
1277	*TET2*	*TET2*: c.4891_dup; p.Y1631L (27%)
2040	*TET2*	*TET2*: c.4402_4405dup; p.Leu1469Glnfs*10 (31%)
2040	*TET2*	TET2c.5356G>T; p.Glu1786* (33%)
2042	*TET2*	*TET2*: c.3594+2dup; p.? (76%)
2049	*TET2*	*TET2*: c.3268_3269del; p.Lys1090Aspfs*13 (33%)
2050	*TET2*	*TET2*: c.3050_3068del19; p.Asp1017Alafs*10 (21%)

2050	*TET2*	*TET2*: c.4639C>T; p.Gln1547* (29%)
2054	*TET2*	*TET2*: c.2185C>T; p. Gln279* (22%)
2079	*TET2*	*TET2*: c.4393C>T; p.Arg1465* (35%)
1255	*TP53*	*TP53*
2057	*TP53*	*TP53*: c:525G>A; Arg175His (5.5%)
2057	*TP53*	*TP53*: c488a>G; Tyr163Cys (7.5%)
1067	*U2AF1*	*U2AF1*: c.101C>T; p.Ser34Phe (26%)
1045	*ZRSR2*	*ZRSR2*: c.106C>T;p.Arg36* (15%)
1045	*ZRSR2*	*ZRSR2*: c.558-1G>C;p.? (60%)
1151	*ZRSR2*	*ZRSR2*: c.348del; p.Glu118Argfs*47 (63%)
1204	*ZRSR2*	*ZRSR2*: c.787G>T; p.Glu263* (52%)

Supplementary Table 4: Cox proportional hazard analysis for myeloid neoplasm free survival (MNFS)

Variable	Hazard ratio	95% CI	*P*-value
**No cytogenetic abnormality (*vs.* abnormal cytogenetics)**	**4.15**	**1.1-15.5**	**0.034**
**Absence of PV in *DTA*-genes (*vs.* PV in *DTA*)**	**8.45**	**0.9-76.9**	**0.06**
PV in *TP53* (*vs.* not)	6.94	0.6-77.7	0.12
**Prior autologous stem cell transplant (*vs.* not)**	3.33	0.64-17.2	0.15
Anemia at t-CC diagnosis (*vs.* not)	5.78	0.73-45.8	0.0967
Prior immunosuppressive therapy (*vs.* not)	0.27	0.03-2.1	0.21
Prior chemotherapy (*vs.* not)	2.44	0.5-11.8	0.27
Neutropenia at t-CC diagnosis (*vs.* not)	0.52	0.13-2.2	0.37
Male gender (*vs.* female)	1.58	0.4-6.3	0.52
Prior radiotherapy (*vs.* not)	1.38	0.35-5.3	0.64
PV in *DNMT3A* (*vs.* not)	1.05	0.11-10.3	0.97

PV – pathogenic variant; DTA – *DNMT3A, TET2*, and *ASXL1* genes; t-CC – therapy-related clonal cytopenia

Supplementary Table 5: Cox proportional hazard analysis for progression free survival (PFS)			
**Variable**	**Hazard ratio**	**95% CI**	***P*-value**
**Abnormal cytogenetic (*vs.* normal cytogenetics)**	**4.18**	**1.4-12.51**	**0.01**
**Prior autologous stem cell transplant (vs. not)**	**5.02**	**1.2-21.07**	**0.03**
**Thrombocytopenia present (*vs.* not)**	**9.1**	**1.18-70.12**	**0.03**
**Anemia present (*vs.* not)**	**8.58**	**1.11-66.21**	**0.04**
**Absence of PV in *DTA*-genes (vs. PV in *DTA*)**	**4.12**	**1.02-16.74**	**0.05**
PV in *TET2* (*vs.* not)	0.24	0.05-1.21	0.08
Prior IST (*vs.* not)	0.34	0.07-1.54	0.16
PV in *RAS* (*vs.* not)	4.58	0.5-41.54	0.18
Neutropenia present (*vs.* not)	0.48	0.15-1.59	0.23
PV in *ASXL1* (*vs.* not)	2.98	0.33-26.8	0.33
Prior chemotherapy (*vs.* not)	1.75	0.54-5.75	0.35
PV in *DNMT3A* (*vs.* not)	0.51	0.06-4.2	0.53
PV in *TP53* (*vs.* not)	1.94	0.23-16.34	0.54
Male gender (*vs.* female)	1.14	0.37-3.51	0.81
Prior radiotherapy (*vs.* not)	0.99	0.33-3.02	0.99

t-CC – therapy-related clonal cytopenia; PV – pathogenic variant; *DTA – DNMT3A, TET2,* or *ASXL1*

**Supplementary Table 6: Cox proportional hazard analysis for overall survival (OS)**			
**Variable**	**Hazard ratio**	**95% CI**	***P*-value**
**Prior autologous stem cell transplant (*vs.* not)**	**8.82**	**1.69-46.13**	**0.01**
**No cytogenetic abnormality (*vs.* abnormal cytogenetics)**	**4.29**	**1.19-15.49**	**0.03**
Anemia at t-CC diagnosis (*vs.* not)	6.47	0.82-51.17	0.08
Thrombocytopenia at t-CC diagnosis (*vs.* not)	5.61	0.71-44.35	0.1
Absence of PV in *DTA*-genes (*vs.* PV in *DTA*)	4.28	0.78-23.62	0.1
PV in *RAS* (*vs.* not)	5.62	0.58-54.78	0.14
PV in *TET2* (*vs.* not)	0.32	0.06-1.77	0.19
Neutropenia at t-CC diagnosis (*vs.* not)	0.36	0.08-1.71	0.2
Prior immunosuppressive therapy (*vs.* not)	0.4	0.08-1.87	0.24
PV in *ASXL1* (*vs.* not)	3.89	0.4-37.85	0.24
Prior radiotherapy (*vs.* not)	1.36	0.39-4.73	0.63
Prior chemotherapy (*vs.* not)	1.25	0.35-4.46	0.73
Male gender (*vs.* female)	1.22	0.31-4.73	0.78

PV – pathogenic variant; DTA – *DNMT3A, TET2*, and *ASXL1* genes; t-CC – therapy-related clonal cytopenia

Supplementary Table 7: Outcomes of patients with clonal cytopenia and the presence of MDS-defining cytogenetics

UPIN	Patient characteristics							Genetic characteristics		Last known phenotype	Time to develop t-MN	Follow-up (months)	Vital status	Primary Cause of death
	Age	Sex	Primary	Chemo	Rad	Auto SCT	IST	Cytogenetics at t-CC diagnosis	Pathogenic variants					
1036	76	F	Lymphoma	Y	Y	N	N	46,XX,del(5)(q13q33),t(10;13)(p10;q10)[3]/46,XX[17]	*ASXL1, TP53*	No t-MN		44	Alive	
1043	78	F	Lymphoma	Y	Y	N	N	45-46,XX,-7,+0-1r[cp2]/46,XX[18]	*TET2, TET2, DNMT3A*	t-MN	43	56	Dead	t-MN
1051	85	F	Thyroid, renal	N	Y	N	N	46,XX,add(7)(q11.2)[2]/46,XX[18]	NA	t-MN	0.9	9	Alive	
1081	66	M	Lymphoma	Y	N	N	N	46,XY,add(5)(q11.2)[1]/45,sl,add(6)(q25),psudic(12;17)(p11.2;p11.2)ins(12;?)(p11.2;?), add(17)(p13)[cp5]/46,XY[14]	*TP53, TP53*	t-MN	14	21	Dead	GvHD
1091	61	F	Lymphoma	Y	N	N	N	46,XX,r(7)[7]/46,XX[13]		t-MN	81	95	Dead	NA
1124	63	M	Lymphoma	Y	Y	N	N	45,X,-Y,add(5)(q11.2)[3]/46,XY[11]	*TP53, TP53*	t-MN	11	13	Dead	Primary
1279	71	F	OLT	N	N	N	Y	45-46,X,add(X)(p11.2),add(2)(q11.2), der(5)t(5;7)(q13;q11.2), add(7)(q32),add(15)(p11.2), ?20[cp11]46,XX[9]	*TP53, TP53*	t-MN	2	23	Dead	NA
1725	69	F	Lymphoma	Y	Y	N	N	44,XX,-5,-7,inv(18)(q21q23)[13]/46,XX[7]	*TP53, TP53*	t-MN	9	9.5	Dead	t-MN
2035	76	F	Lymphoma, Breast	Y	N	N	N	45,XX,+5,add(5)(q11.2)x2,-7,-13,der(20)t(13;20)(q14;q13.1)[15]/46,XX[5]	*TP53*	t-MN	3	5	Dead	t-MN
2041	81	F	Lymphoma, Breast	Y	N	N	N	46,XX,add(5)(q11.2)[3]/45,XX,der(5;15)(p10;q10), add(13)(p13)[12]/46,XX[5]	NA	No t-MN		12	Dead	Primary
2046	64	F	Lung	Y	Y	N	N	45,XX,add(5)(q11.2),-18[cp5]/ 46,XX, +1, der(1;5)(q10;p10)[cp4]/46,XX, add(5)(q22)[2]/ 43,XX,add(5)(q11.2),-7,del(7)(q22),-8, -17[1]/ 46,XX[8]	*TP53, TP53*	t-MN	3	7	Dead	t-MN
2048	85	M	Lymphoma	Y	N	N	N	46,XY,del(20)(q11.2q13.3)[10]/56-65,sl,+1,+5,del(7)(q11.2q22),+9,+10,+11,+15,+22[cp2]/46,XY, del(7)(p11.2p15)[2]/46,XY[6]	*ASXL1, IDH1, SRSF2*	No t-MN		0.7	Dead	Sepsis

MDS – myelodysplastic syndrome; UPIN – unique patient identification number; F – female; M – Male; chemo – chemotherapy; rad – radiation therapy; SCT – stem cell transplant; IST – immunosuppressive therapy; Y – yes; N – No; t-MN – therapy-related myeloid neoplasm; GVHD – graft-*vs.*-host disease; OLT – orthotopic liver transplant; NA – not available.

(B) Supplementary Figures

Supplementary figure 1: Clinical and genetic characteristics of t-MDS (cyto) patients.

Supplementary figure 2: (i) Time to develop morphologic evidence of t-MN for patients with therapy-related clonal cytopenia (t-CC) and those presenting with MDS-defining cytogenetic abnormalities in the absence of morphological evidence of a myeloid neoplasm [t-MDS(cyto.)]; and (ii) overall survival (OS) in patients with t-CC compared to t-MDS(cyto).

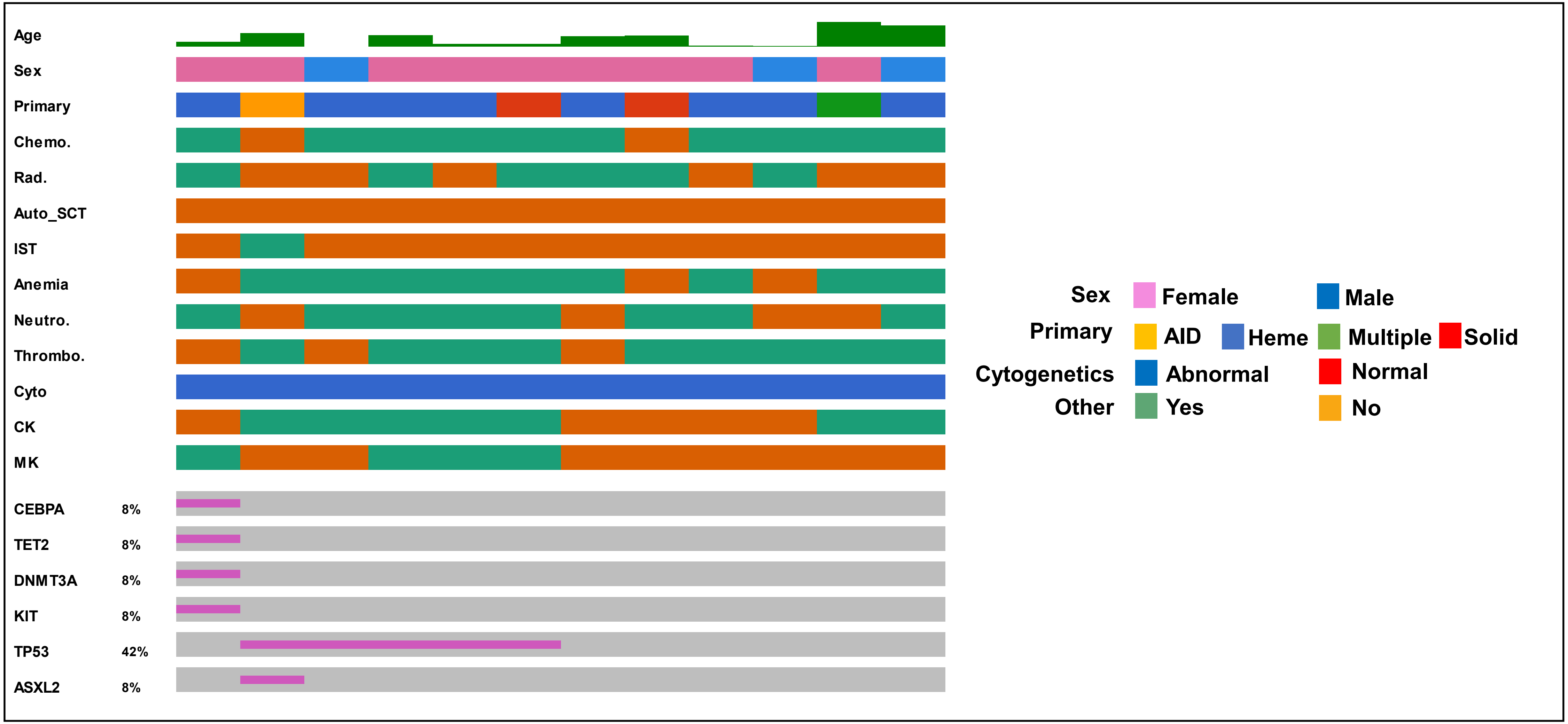

Supplementary Figure 1: Clinical and genetic characteristics of t-MDS (cyto) patients.

AID – autoimmune disease, Heme – primary hematological malignancy; Solid – primary solid tumor; Chemo. – chemotherapy; Rad. – radiation; Auto SCT – autologous stem cell transplant; IST – immunosuppressive therapy; Neutro. – neutropenia; Thrombo. – thrombocytopenia, CK – complex karyotype; MK – monosomal karyotype.

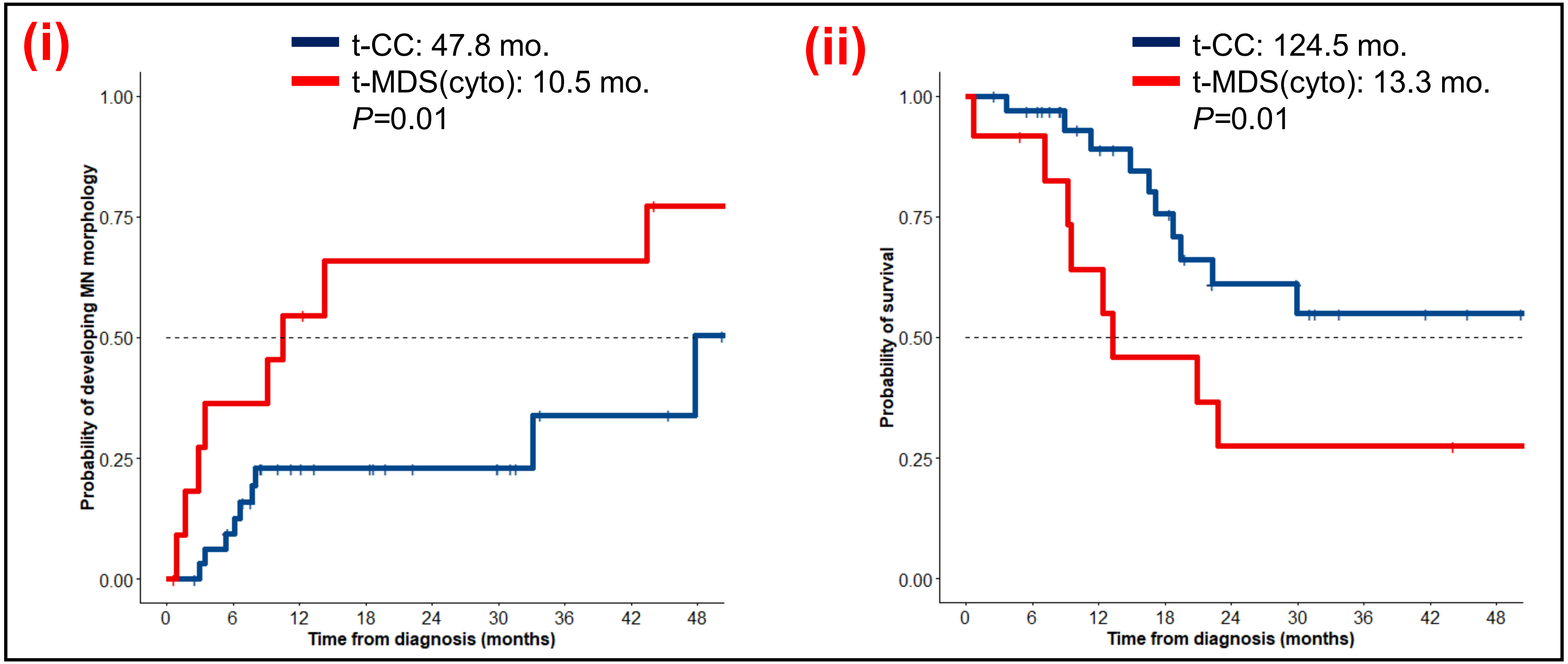

Supplementary Figure 2 (i) Time to develop morphologic evidence of t-MN for patients with therapy-related clonal cytopenia (t-CC) and those presenting with MDS-defining cytogenetic abnormalities in the absence of morphological evidence of a myeloid neoplasm [t-MDS(cyto)]; and (ii) overall survival (OS) in patients with t-CC compared to t-MDS(cyto).
